# Bloodstream Infections in Patients with Malignancy: Microbiological Epidemiology, Antimicrobial Resistance, and Factors Associated with Mortality in a Nine-Year Single-Center Retrospective Cohort

**DOI:** 10.3390/diagnostics16203314

**Published:** 2026-10-12

**Authors:** Cihan Yeşil, Cemile Arslan Yeşil, Ferhat Yılmaz, Özgür Tanrıverdi, Gökhan Pektaş

**Affiliations:** 1 Department of Infectious Diseases and Clinical Microbiology, Muğla Training and Research Hospital, 48000 Muğla, Türkiye; arslancemile9@gmail.com; 2 Department of Internal Medicine, Muğla Training and Research Hospital, 48000 Muğla, Türkiye; yilmaz.ferhat@yahoo.com; 3 Division of Oncology, Muğla Training and Research Hospital, 48000 Muğla, Türkiye; ozgurtanriverdi@mu.edu.tr; 4 Division of Hematology, Muğla Training and Research Hospital, 48000 Muğla, Türkiye; gokhanpektas@mu.edu.tr

**Keywords:** malignancy, bloodstream infection, bacteremia, antimicrobial resistance, neutropenia, mortality

## Abstract

**Background/Objectives:** Bloodstream infections (BSIs) are a major cause of morbidity and mortality among patients with malignancy because of disease-related immune dysfunction, intensive chemotherapy, neutropenia, and frequent exposure to invasive procedures. This study aimed to characterize the microbiological profile and antimicrobial resistance patterns of BSIs in patients with malignancy and to identify clinical and laboratory factors associated with in-hospital mortality, explicitly treating ICU admission as an associated marker of critical illness rather than a predictor. **Methods:** This retrospective study included 191 clinically significant BSI episodes recorded between 2016 and 2025; record review confirmed that each included episode represented a different patient. Demographic characteristics, underlying diagnoses, comorbidities, chemotherapy history, catheter status, infection source, hematological and biochemical parameters, bloodstream pathogen category, antimicrobial susceptibility patterns, ward and intensive care unit (ICU) lengths of stay, and in-hospital mortality were evaluated. Categorical variables were compared using the chi-square or Fisher’s exact test, while continuous variables were analyzed using the *Mann–Whitney U* test. Multivariable logistic regression analysis was performed to identify factors independently associated with in-hospital mortality. **Results:** Of the 191 episodes, 96 (50.3%) occurred in female patients and 95 (49.7%) in male patients. Acute leukemia was the most common underlying diagnosis (*n* = 70, 36.6%). Blood cultures yielded Gram-negative organisms in 110 episodes (57.6%), Gram-positive organisms in 58 (30.4%), fungi in 19 (9.9%), and polymicrobial growth in 4 (2.1%). Among 168 episodes with interpretable bacterial antimicrobial susceptibility results, 99 (58.9%) met the study-defined composite resistance category was not equivalent to multidrug resistance. An indwelling catheter was present in 27 episodes (14.1%), ICU admission occurred in 25 (13.1%), and severe neutropenia (<500 cells/µL) was documented in 72 (37.7%). The median ward length of stay was 8.0 days (interquartile range, 5.0–14.0 days). In-hospital mortality occurred in 39 episodes (20.4%). Patients who died had significantly lower neutrophil, platelet, lymphocyte, and albumin levels and significantly higher urea and C-reactive protein levels than survivors (*p* < 0.01). In multivariable analysis, ICU admission (adjusted odds ratio [aOR], 18.94; 95% confidence interval [CI], 3.49–102.82; *p* < 0.001) and severe neutropenia (aOR, 6.06; 95% CI, 2.13–17.23; *p* < 0.001) were associated with in-hospital mortality. However, the wide confidence intervals, particularly for ICU admission, indicate substantial uncertainty in the magnitude of these associations. **Conclusions:** These estimates should therefore be interpreted cautiously, especially because the timing of ICU admission relative to BSI onset could not be established. Gram-negative organisms predominated among BSI episodes in patients with malignancy, and study-defined resistance phenotypes were frequent but heterogeneous. Severe neutropenia and ICU admission—analyzed specifically as a concurrent marker of critical illness rather than a predictor due to unestablished temporal sequence relative to BSI onset—were associated with in-hospital mortality. These estimates should be interpreted cautiously. Continuous surveillance of local pathogen distributions and resistance phenotypes, early identification of high risk patients, and institution-specific selection of empirical antimicrobial therapy may contribute to improved clinical outcomes.

## 1. Introduction

The global burden of malignancy has increased steadily over recent decades, while advances in chemotherapy, targeted therapy, immunotherapy, and supportive care have substantially improved patient survival [1]. However, these developments have also resulted in a growing population exposed to prolonged or recurrent immunosuppression. Bloodstream infections (BSIs) are among the most serious infectious complications in patients with malignancy and are associated with treatment interruption, prolonged hospitalization, ICU admission, and increased mortality [2,3]. The increased susceptibility of patients with malignancy to BSI is attributable to the combined effects of the underlying disease and anticancer treatment. Bone marrow infiltration, impaired cellular and humoral immunity, chemotherapy-induced mucosal injury, corticosteroid exposure, and repeated administration of *broad-spectrum* antimicrobial agents contribute to the development of infection [3,4]. Neutropenia, particularly when severe or prolonged, remains one of the most important predisposing conditions. Because the inflammatory response may be attenuated in neutropenic patients, infection can progress rapidly despite limited clinical findings [5,6]. Central venous catheters, which are commonly used for chemotherapy, transfusion, parenteral nutrition, and supportive treatment, provide an additional portal of entry for microorganisms and represent an important source of healthcare-associated BSI [7]. The microbiological epidemiology of BSI in patients with malignancy has changed over time and varies considerably across institutions and geographical regions. Although Gram-positive organisms became increasingly prominent with the widespread use of central venous catheters and fluoroquinolone prophylaxis, recent studies have demonstrated a renewed predominance of Gram-negative bacilli in many oncology and hematology populations. Frequently reported Gram-negative pathogens include *Escherichia coli*, *Klebsiella* spp., *Pseudomonas aeruginosa*, and *Acinetobacter baumannii* [8,9]. Gram-positive organisms, particularly coagulase-negative staphylococci, *Staphylococcus aureus*, and enterococci, remain clinically relevant, especially in catheter-associated infections [3,7]. Fungal and polymicrobial BSIs are less common but may be associated with delayed effective therapy and unfavorable clinical outcomes, as previously described in dedicated polymicrobial cohort evaluations in hematologic malignancy [10]. Antimicrobial resistance represents an increasingly important challenge in the management of BSI among patients with malignancy. Multidrug-resistant Gram-negative bacilli, methicillin-resistant staphylococci, and vancomycin-resistant *enterococci* have become more frequently reported in this vulnerable population [11,12]. Previous antimicrobial exposure, recurrent hospitalization, prolonged neutropenia, invasive devices, and colonization with resistant organisms may further increase this risk [12,13]. Recent studies have documented high rates of extended-spectrum β-lactamase production and carbapenem resistance among Gram-negative isolates obtained from patients with cancer [13,14]. These resistance patterns restrict empirical treatment options and increase the likelihood of initially inappropriate antimicrobial therapy. Prompt administration of effective empirical antimicrobial therapy is essential because delays or discordant initial treatment may adversely affect survival. Current recommendations therefore emphasize early clinical risk assessment, timely blood culture collection, rapid initiation of broad-spectrum empirical therapy in clinically unstable patients, and subsequent modification according to microbiological findings and antimicrobial susceptibility results [15]. A large multicenter analysis demonstrated that empirical therapy discordant with in-vitro susceptibility was independently associated with increased in-hospital mortality [16]. Nevertheless, excessively broad empirical treatment may promote further resistance, highlighting the need to balance adequate early coverage with antimicrobial stewardship. Clinical outcomes following BSI are determined by the interaction of host-related, malignancy-related, microbiological, and treatment-related factors. Severe and prolonged neutropenia remains one of the most critical determinants of invasive bacterial and fungal bloodstream infections in patients with hematological malignancies, necessitating adherence to updated risk-stratified management strategies and evidence-based guidelines [17,18,19]. Profound neutropenia, organ dysfunction, intensive care requirement, invasive catheter use, resistant pathogens, and inadequate empirical therapy have been associated with poor outcomes [9,11]. Laboratory markers reflecting inflammation, nutritional status, renal function, and hematopoietic reserve may also contribute to early risk stratification. However, the prognostic importance of these factors differs between centers because of variations in patient characteristics, cancer treatments, local pathogen distributions, and antimicrobial resistance patterns. Contemporary institution-specific data are therefore necessary to guide empirical therapy and identify patients at increased risk of adverse outcomes. The present study aimed to retrospectively characterize BSIs in patients with malignancy, describe the distribution and temporal changes in bloodstream pathogens and their antimicrobial susceptibility patterns, and evaluate the relationships of demographic, clinical, treatment-related, and laboratory variables with pathogen characteristics, hospital and intensive care unit lengths of stay, and in-hospital mortality.

## 2. Methods

### 2.1. Study Design and Setting

This single-center, retrospective observational study evaluated 1344 suspected bloodstream infection episodes among adult patients diagnosed with malignancy at Muğla Sıtkı Koçman University Muğla Training and Research Hospital between January 2016 and January 2025. Of these, 475 episodes yielded positive blood cultures, and 191 cases were ultimately included in the cohort (Figure 1). Annual trend analyses (Figure 2 and Figure 3, and Supplementary Table S2) are restricted to full calendar years (2016–2024), as January 2025 contributed only two episodes; all 191 episodes across the complete timeframe are included in overall cohort analyses. Patients were identified hospital-wide across all inpatient services, including the Departments of Hematology, Medical Oncology, Internal Medicine, and Intensive Care Units. The study was designed and reported in accordance with the Strengthening the Reporting of Observational Studies in Epidemiology (STROBE) recommendations [20]. All eligible BSI episodes recorded during the study period were included; therefore, no formal a priori sample-size calculation was performed. The analytical unit was an individual clinically significant BSI episode rather than an isolated laboratory result. Review of the available records confirmed that each of the 191 included episodes represented a different patient, including episodes involving coagulase-negative staphylococci; observations were therefore treated as independent. Patient records, hospital information systems, and microbiology laboratory databases were retrospectively reviewed using a standardized data-collection framework.

### 2.2. Study Population and Episode Selection

During the study period, 1344 suspected infection episodes screened hospital-wide across hematology, oncology, internal medicine, and intensive care services were evaluated. Microbial growth was detected in 475 blood-culture episodes. Case identification was performed through systematic review of microbiology laboratory records and electronic hospital health system alerts. Microbiological definitions were based on standard surveillance criteria: a true BSI episode required the isolation of a recognized pathogen from blood cultures accompanied by clinical signs of infection, whereas skin commensals (such as coagulase-negative staphylococci) required isolation from at least two separate blood culture sets to rule out contamination. Of these, 284 were classified as contaminants following clinical and microbiological review and were excluded. The final analytical cohort consisted of 191 clinically significant BSI episodes. The inclusion criteria were as follows: (1) age ≥18 years; (2) a diagnosis of a hematological or solid malignancy; (3) at least one clinically significant positive blood culture obtained during the study period; and (4) availability of clinical outcome data for the index hospitalization. Blood cultures considered representing contamination rather than true infection were excluded. Records with missing values for individual laboratory or clinical variables were retained in the overall cohort and included in the relevant analyses using an available-case approach.

### 2.3. Definitions

A clinically significant BSI was defined as the isolation of a recognized bacterial or fungal pathogen from at least one blood-culture set in the presence of compatible clinical findings. For microorganisms commonly associated with skin contamination, including coagulase-negative *staphylococci*, viridans group *streptococci*, and *Micrococcus* spp., clinical significance required recovery of the same organism from at least two blood cultures obtained on separate occasions, together with compatible signs of infection. These definitions were based on established bloodstream-infection surveillance criteria [21]. A polymicrobial BSI was defined as the recovery of two or more clinically significant microorganisms from blood cultures obtained within the same infection episode. Severe neutropenia was defined as an absolute neutrophil count below 500 cells/µL at the time of BSI. Catheter exposure was defined as the presence of an intravascular catheter when the index blood culture was obtained. A catheter-related infection was recorded when the catheter was documented as the presumed infection source on the basis of clinical and microbiological assessment. The infection source was classified as, primary bloodstream focus, catheter-related infection, respiratory tract infection, or urinary tract infection. In-hospital mortality was defined as death from any cause during the index hospitalization following the diagnosis of BSI. Ward and intensive care unit (ICU) lengths of stay were calculated in days. ICU admission was defined by a recorded ICU stay during the index hospitalization. The dataset did not identify the patient’s care location at BSI onset or whether ICU admission preceded, coincided with, or followed the diagnosis of BSI; ICU admission was therefore analyzed as an associated marker rather than a temporally ordered exposure.

### 2.4. Data Collection

The following demographic and clinical variables were collected: calendar year of the positive blood culture, sex, underlying malignancy category, comorbidities, number of chemotherapy courses, most recent chemotherapy regimen, history of surgery, ward length of stay, ICU admission and length of stay, catheter status, catheter duration, and presumed infection source. Underlying diagnoses were categorized as acute leukemia, lymphoma, plasma-cell disorder, chronic leukemia, or other hematological/solid malignancy. Comorbidities included diabetes mellitus, chronic obstructive pulmonary disease, chronic kidney disease, hypertension, coronary artery disease, atrial fibrillation, chronic liver disease, additional oncological malignancy, and neurological disease. The most recent anticancer treatment was categorized as supportive or palliative treatment, targeted therapy, a multiple-myeloma regimen, a hypomethylating-agent regimen, a standard combination regimen, or a high-intensity regimen. Laboratory values recorded at the time of BSI included absolute neutrophil, lymphocyte, monocyte, eosinophil, and platelet counts; hemoglobin, urea; creatinine; alanine aminotransferase, aspartate aminotransferase; total protein; globulin; albumin; lactate dehydrogenase (LDH); C-reactive protein; procalcitonin; and erythrocyte sedimentation rate. The following derived inflammatory indices were also evaluated: LDH-to-lymphocyte ratio, eosinophil-to-lymphocyte ratio, lymphocyte-to-monocyte ratio, neutrophil-to-albumin ratio, platelet-to-lymphocyte ratio, and neutrophil-to-lymphocyte ratio. Before statistical analysis, the dataset was assessed for coding consistency, impossible values, missing observations, and agreement between component laboratory measurements and derived indices. The original clinical data were not overwritten during the quality-control process. Data regarding specific empirical antimicrobial regimens, the appropriateness of initial empirical therapy relative to in vitro susceptibility results, and routine antibacterial prophylaxis exposure were not systematically captured in the medical records and were therefore not available for analysis.

### 2.5. Microbiological Assessment

Blood cultures were processed in the hospital microbiology laboratory using routine automated culture methods. Identification and antimicrobial susceptibility testing of recovered microorganisms were performed using the laboratory procedures and European Committee on Antimicrobial Susceptibility Testing breakpoints applicable during the relevant study year. Bloodstream pathogens were grouped as Gram-positive bacteria, Gram-negative bacteria, fungi, or polymicrobial growth. Antimicrobial susceptibility findings were categorized according to the prespecified study coding system. Methicillin-resistant coagulase-negative staphylococci, methicillin-resistant *S. aureus*, resistant Gram-negative organisms, and ampicillin-resistant organisms were classified as resistant. Methicillin-susceptible coagulase-negative staphylococci, methicillin-susceptible *S. aureus*, susceptible Gram-negative organisms, and ampicillin-susceptible organisms were classified as susceptible. This heterogeneous binary composite was used to preserve analyzable group sizes. Species -and phenotype-specific comparisons were explored but were generally underpowered and did not yield statistically significant associations because of small subgroup counts. Fungal and polymicrobial episodes were not included in the denominator when calculating the composite proportion of bacterial resistance. For patients with catheter cultures, catheter-derived organism groups and susceptibility patterns were compared with the corresponding bloodstream findings. Calendar-year distributions were examined to evaluate temporal changes in bloodstream pathogen categories and resistance patterns.

### 2.6. Study Outcomes

The primary outcome was all cause in-hospital mortality following the index BSI. Secondary outcomes included:

- Distribution of bloodstream pathogens and antimicrobial susceptibility patterns;
- Changes in pathogen and resistance distributions between 2016 and 2025;
- Associations between clinical, treatment-related, and laboratory variables and bloodstream pathogen or resistance category;
- Ward and ICU lengths of stay;
- Relationships between catheter and blood-culture pathogen categories and susceptibility patterns;
- Clinical and laboratory factors associated with in-hospital mortality.

### 2.7. Statistical Analysis

Categorical variables were summarized as frequencies and percentages. Continuous variables were examined for distributional characteristics and, because most laboratory and length-of-stay variables were non-normally distributed, were summarized as medians with interquartile ranges (IQRs). Missing data were not imputed; the number of available observations was retained for each analysis. Categorical variables were compared using *Pearson’s* chi-square test or *Fisher’s* exact test when expected cell counts were less than 5. Continuous variables were compared between survivors and non-survivors using the *Mann–Whitney U* test. Associations involving pathogen class, susceptibility category, diagnosis group, chemotherapy regimen, and infection source were evaluated using contingency-table analyses. Temporal changes in annual resistance proportions were explored using *Spearman’s* rank-correlation coefficient. Continuous outcomes were compared across anticancer-treatment categories using the *Kruskal–Wallis* test; while sparse multi-category contingency tables were evaluated using the *Fisher-Freeman-Halton* exact test with *Monte Carlo* estimation. A multivariable binary logistic regression model was constructed to identify factors independently associated with in-hospital mortality. All 191 BSI episodes—including fungal (*n* = 19) and polymicrobial (*n* = 4) cases-were retained in the analysis. To prevent severe model over fitting, parameter inflation, and unstable estimates in accordance with the events-per-variable (EPV) rule of thumb-given the occurrence of 39 in-hospital deaths—the number of candidate predictors was strictly limited a priori to four variables (ICU admission, catheter presence, severe neutropenia, and male sex). Candidates were selected on the basis of established clinical relevance, prognostic plausibility, and data completeness. Downstream laboratory biomarkers of acute organ dysfunction and systemic inflammatory response (e.g., CRP, blood urea nitrogen, albumin, and platelet count) were intentionally excluded from the multivariable model to avoid severe multicollinearity and over-adjustment bias. The model was designed as a parsimonious exploratory evaluation rather than a validated risk prediction tool. Given that the exact temporal relationship between ICU stay and BSI onset could not be established, ICU admission was treated strictly as an associated marker of critical illness recorded during index hospitalization rather than a prospective predictor. To address potential temporal confounding, reverse causality, or routine postoperative ICU transfers among surgical patients (22.5% of the cohort), a secondary sensitivity multivariable logistic regression model was performed excluding the ICU admission variable. Results are expressed as adjusted odds ratios (aORs) with 95% confidence intervals (CIs). All statistical tests were two-sided, and a *p* value < 0.05 was considered statistically significant. Because secondary outcomes were exploratory, no formal adjustment for multiple testing was applied. Statistical analyses were performed using Python version 3.12.13 with pandas version 2.2.3, NumPy version 2.3.5, and SciPy version 1.17.0.

### 2.8. Ethical Considerations

The study protocol was approved by the Muğla Sıtkı Koçman University Medical Sciences Ethics Committee (approval date: 26 February 2025; decision number: 54; protocol number: 250041). The requirement for written informed consent was waived due to the retrospective design of the study and the use of de-identified clinical data. The study was conducted in accordance with the principles of the Declaration of Helsinki, applicable national regulations concerning medical research, and patient data confidentiality.

## 3. Results

### 3.1. Study Cohort and Clinical Profile

During the 2016–2025 study period, 1344 suspected infection episodes were evaluated. Blood cultures were positive in 475 episodes; after exclusion of 284 cultures classified as contaminants, 191 clinically significant bloodstream infection episodes constituted the analytic cohort (Figure 1). Of these episodes, 95 (49.7%) occurred in male patients. Acute leukemia was the most frequent underlying diagnostic category (70/191, 36.6%), followed by plasma cell disorders (38/191, 19.9%) and lymphoma (35/191, 18.3%). A central venous catheter was present in 27 episodes (14.1%), 25 episodes (13.1%) required intensive care unit (ICU) admission, and severe neutropenia (<500 cells/µL) was documented in 72 (37.7%). The median ward length of stay was 8 days (interquartile range [IQR], 5.0–14.0) (Table 1).

### 3.2. Microbiological Characteristics

Gram-negative organisms accounted for 110 episodes (57.6%), Gram-positive organisms for 58 (30.4%), fungi for 19 (9.9%), and polymicrobial growth for 4 (2.1%) (Table 2). Among 168 bacterial episodes with interpretable susceptibility profiles, 99 (58.9%) met the study-defined composite resistance criteria. Because combining distinct resistance mechanisms across diverse Gram-positive and Gram-negative organisms introduces significant clinical heterogeneity, this variable was designated strictly as an exploratory composite endpoint rather than a standardized multidrug-resistance metric. Species- and phenotype-specific subgroup analyses were constrained by small sample sizes across individual pathogen subsets and did not demonstrate statistically significant associations with in-hospital mortality. Neither pathogen class (*p* = 0.296) nor the composite bacterial resistance category (*p* = 0.559) differed significantly by survival status. The distribution of infection focus differed between survivors and non-survivors (overall *p* = 0.038), although individual focus categories were sparse. Catheter cultures were positive in 4 of 27 catheterized episodes (14.8%); in all four, both pathogen class and susceptibility category were concordant with the corresponding blood isolate (Supplementary Table S1). Annual pathogen composition is shown in Figure 2.

### 3.3. Laboratory Findings

Compared with survivors, non-survivors had lower median neutrophil (120 vs. 2200 cells/µL; *p* < 0.001), platelet (17,000 vs. 44,000 cells/µL; *p* < 0.001), lymphocyte (270 vs. 710 cells/µL; *p* = 0.001), monocyte (80 vs. 350 cells/µL; *p* = 0.004), and eosinophil counts (0 vs. 10 cells/µL; *p* < 0.001). Non-survivors also had higher urea (55.6 vs. 39.7 mg/dL; *p* < 0.001) and C-reactive protein concentrations (193.8 vs. 100.2 mg/L; *p* = 0.002), together with lower total protein (50.1 vs. 56.3 g/L; *p* < 0.001) and albumin levels (27.6 vs. 31.0 g/L; *p* < 0.001). Hemoglobin, creatinine, and procalcitonin did not differ significantly between groups. Cell-count-derived ratios involving neutrophils were also lower among non-survivors, consistent with the greater burden of profound neutropenia in this group (Table 3).

### 3.4. Mortality and Multivariable Analysis

Overall in-hospital mortality was 20.4% (39/191). The most recent anticancer-treatment category was not associated with in-hospital mortality (exact *p* = 0.192), ICU admission (exact *p* = 0.717), ward length of stay (*Kruskal–Wallis p* = 0.264), or ICU length of stay among ICU-admitted episodes (*Kruskal–Wallis p* = 0.192). In the complete cohort multivariable regression model (*n* = 191, incorporating all 39 in-hospital deaths), fungal and polymicrobial episodes were fully retained alongside Gram-positive and Gram-negative bacterial cases. In unadjusted analyses, mortality was associated with male sex (odds ratio [OR], 2.41; 95% confidence interval [CI], 1.15–5.03; *p* = 0.020), catheter presence (OR, 21.8; 95% CI, 8.15–58.37; *p* < 0.001), ICU admission (OR, 43.17; 95% CI, 13.32–139.90; *p* < 0.001), and severe neutropenia (OR, 5.35; 95% CI, 2.49–11.49; *p* < 0.001). In the multivariable logistic regression model, ICU admission (adjusted OR [aOR], 18.94; 95% CI, 3.49–102.82; *p* < 0.001) and severe neutropenia (aOR, 6.06; 95% CI, 2.13–17.23; *p* < 0.001) remained independently associated with mortality. The associations for catheter presence (aOR, 3.59; 95% CI, 0.68–18.89; *p* = 0.132) and male sex (aOR, 1.11; 95% CI, 0.40–3.06; *p* = 0.840) were attenuated after adjustment (Table 4 and Figure 3). In a sensitivity multivariable model excluding ICU admission, severe neutropenia maintained a robust independent association with mortality (aOR, 4.12; 95% CI, 1.85–9.18; *p* < 0.001), while catheter presence (aOR, 2.45; 95% CI, 0.88–6.82; *p* = 0.086) and male sex (aOR, 1.35; 95% CI, 0.58–3.14; *p* = 0.485) remained non-significant. The model was significant overall (likelihood-ratio chi-square = 74.32, 4 df; *p* < 0.001) and had an apparent area under the receiver operating characteristic curve of 0.858. The wide confidence intervals, particularly for ICU admission (aOR, 18.94; 95% CI, 3.49–102.82) and catheter presence (aOR, 3.59; 95% CI, 0.68–18.89), indicate substantial uncertainty in the magnitude of these associations. The large crude odds ratios for ICU admission (OR, 43.17; 95% CI, 13.32–139.90) and catheter presence (OR, 21.80; 95% CI, 8.15–58.37) should also be interpreted cautiously in view of the limited number of deaths and the possibility of sparse-data effects. Because no internal validation (such as bootstrapping or cross-validation) was performed, the reported AUC of 0.858 represents apparent discrimination and may be subject to optimistic bias.

### 3.5. Temporal Patterns

The annual number of clinically significant bloodstream infection episodes ranged from 16 to 25. Gram-negative organisms predominated in eight of the nine study years (Figure 2). The annual proportion meeting the study-defined composite bacterial resistance category ranged from 50.0% in 2020 to 73.3% in 2023, without evidence of a monotonic temporal trend (*Spearman* rho = 0.35; *p* = 0.356). Annual in-hospital mortality ranged from 9.1% in 2021 to 37.5% in 2024 and likewise showed no monotonic trend (*Spearman* rho = 0.03; *p* = 0.932) (Figure 4; detailed annual breakdowns are provided in Supplementary Table S2). An exploratory comparison of in hospital mortality during versus outside the COVID-19 pandemic period did not identify a statistically significant difference.

## 4. Discussion

In this nine-year retrospective cohort of 191 clinically significant BSI episodes in patients with malignancy, resistance—as defined by our study criteria—was detected in more than half of the bacterial episodes with interpretable susceptibility data. The most consequential signals were markers of clinical severity and host vulnerability: ICU admission (representing a marker of critical illness) and severe neutropenia remained independently associated with mortality after adjustment, whereas sex and catheter presence did not retain statistical significance. Because ICU timing relative to BSI onset was unavailable and several confidence intervals were wide, these findings describe associations rather than temporal or causal effects. Overall, the results position BSI in cancer as an interaction between pathogen ecology, treatment constraints, and the patient’s physiological reserve rather than as an outcome determined by organism class or resistance status alone. The predominance of Gram-negative organisms (57.6%) is consistent with the re-emergence of Gram-negative BSI reported in contemporary haematology populations. The Italian multicentre prospective survey by Trecarichi et al., demonstrated the major contribution of Gram-negative pathogens and the clinical importance of antimicrobial resistance in haematological malignancy, while Cai et al., documented dynamic shifts in pathogen distribution across recent treatment eras [22,23]. Our observation that Gram-negative organisms were the leading group in eight of nine study years therefore appears clinically plausible and locally important. It supports maintaining reliable anti-Gram-negative coverage in the initial management of unstable patients, while allowing local surveillance not historical assumptions about Gram-positive predominance to guide empirical choices. The study-defined resistance composite was frequent: 58.9% of the 168 susceptibility-interpretable bacterial episodes met its criteria. This percentage is not an MDR rate. It combines organism-specific phenotypes, including methicillin resistance among staphylococci, ampicillin resistance among enterococci, and relevant beta-lactam or carbapenem resistance among Gram-negative organisms. Longitudinal surveillance in haematological malignancy has shown the emergence of resistant bloodstream pathogens, and a recent multicentre cancer cohort confirmed marked heterogeneity in resistance mechanisms and clinical impact across institutions [24,25]. Choi et al., likewise demonstrated that resistance among isolates from patients with haematologic disease is shaped by organism type and healthcare related exposures [26]. Phenotype-specific analyses in the present cohort were generally inconclusive because subgroup counts were small; accordingly, the broader susceptible-versus-resistant comparison was used to preserve analyzable sample size. The composite category was not significantly associated with mortality, but this should not be interpreted as evidence that resistance is clinically inconsequential. In particular, the lack of a statistically significant association between the composite resistance category and in-hospital mortality should be interpreted with caution. Information on initial empirical antimicrobial therapy—specifically whether empirical coverage was concordant or discordant with susceptibility testing—and routine antibacterial prophylaxis was not available in our dataset. Empirical therapy appropriateness is a well-established driver of clinical outcomes in bloodstream infections, and resistance frequently impacts mortality indirectly through delays in effective therapy. The absence of these treatment-related variables precludes a direct evaluation of therapeutic delay as a mediating pathway between antimicrobial resistance and mortality. A notable limitation of our microbiological evaluation is the use of a composite bacterial resistance variable. Merging distinct phenotypes-such as methicillin resistance in staphylococci and beta-lactam or aminoglycoside resistance in Gram-negative bacilli-into a single binary category creates clinical heterogeneity and limits direct clinical comparison between ‘resistant’ and ‘susceptible’ infections. Accordingly, this composite metric should be viewed solely as an exploratory signal. Crucially, the lack of significant association between this composite resistance category and mortality (*p* = 0.559) highlights that in this severely immunocompromised hematology cohort, underlying host vulnerabilities—specifically profound neutropenia and critical clinical deterioration—serve as the dominant determinants of clinical outcome rather than isolated bacterial resistance profiles. Empirical treatment appropriateness and time to active therapy were unavailable, and the number of deaths limited precision. Resistance may affect outcome chiefly through delayed effective therapy, a pathway that could not be directly tested here. The overall mortality of 20.4% falls within the broad range reported in contemporary cancer-associated BSI cohorts, although comparisons are sensitive to case mix, outcome window, infection source, and the proportion of patients with acute leukaemia or critical illness. In a Turkish cohort of febrile neutropenic patients, Gram-negative resistance profiles were associated with adverse outcomes, whereas a separate Turkish study identified clinical status and infection-related characteristics as important mortality determinants [27,28]. The present findings extend this evidence by showing that, after simultaneous adjustment, ICU admission and severe neutropenia accounted for much of the observed mortality gradient. The loss of the crude sex association after adjustment further suggests that sex was acting as a marker of differences in severity or case composition rather than as an independent biological determinant.

ICU admission had the largest adjusted association with death (aOR, 18.94), but its confidence interval was wide and the timing of ICU care relative to BSI onset was unavailable. Some patients may have been in the ICU before BSI recognition, whereas others may have been transferred during subsequent deterioration. ICU admission should therefore be interpreted as a marker of critical illness recorded during the index hospitalization, not as a temporally ordered or causal exposure. Furthermore, as 22.5% of the study cohort had a surgical history, a subset of ICU admissions may have reflected planned postoperative monitoring rather than infection-driven sepsis decompensation. Crucially, our sensitivity analysis excluding ICU admission demonstrated that severe neutropenia retained its strong, independent prognostic association with mortality (aOR = 4.12, 95% CI: 1.85–9.18; *p* < 0.001), confirming that host immunological vulnerability functions as a genuine baseline risk predictor regardless of critical care disposition. The result is compatible with the binational cohort reported by MacPhail et al., in which outcomes of ICU-treated sepsis in patients with haematological malignancy improved over time but remained worse than in patients without malignancy [29]. Clinically, the association supports close monitoring and prompt management of patients with physiological instability, while its magnitude should not be generalized beyond this cohort. Severe neutropenia was independently associated with mortality (aOR, 6.06) and was accompanied by markedly lower neutrophil, lymphocyte, monocyte, and platelet counts among non-survivors. Nevertheless, the literature does not support treating neutropenia as a universally adverse prognostic factor in every sepsis population. In a nationwide cohort of cancer patients with septic shock, Kim et al., found that neutropenia was not associated with increased mortality after adjustment [30]. This apparent discrepancy may reflect differences in population definition, timing, residual confounding, and the distinction between isolated neutropenia and broader marrow failure. In our cohort, severe neutropenia may have captured both impaired innate immune defence and the intensity or activity of the underlying malignancy. Furthermore, because our cohort spans a heterogeneous spectrum of malignancies—ranging from acute leukemias to solid tumors—variations in baseline bone marrow reserve, chemotherapy intensity, and immunosuppression duration may confound observed cell count differences between survivors and non-survivors. Differences in blood cell parameters (Table 2) likely reflect the combined burden of underlying disease biology and treatment intensity alongside the host response to sepsis. Prompt recognition and tailored management of cancer-related neutropenia are essential to optimize therapeutic outcomes and mitigate BSI-associated mortality in high-risk hematology populations [17,18,19]. It should consequently be viewed as a high risk contextual marker rather than a reason to limit intensive treatment. Catheter presence showed a very strong crude association with mortality but a smaller, non-significant adjusted estimate (aOR, 3.59) with a wide confidence interval. This attenuation implies substantial overlap with ICU admission, neutropenia, treatment intensity, or other unmeasured severity factors. Only four catheter cultures were positive, and all four paired catheter and blood isolates were concordant by organism class and susceptibility category. Although this internal concordance supports a catheter-related origin in those episodes, the small number precludes reliable estimates of diagnostic performance or catheter-attributable mortality. The clinically appropriate interpretation is therefore that intravascular devices identify a vulnerable subgroup and require systematic source assessment; these data do not establish the catheter itself as an independent cause of death. The magnitude of the association between ICU admission and mortality should be interpreted cautiously. Although the adjusted association was statistically significant, the wide confidence interval indicates that its magnitude was estimated imprecisely. The large crude odds ratios for ICU admission and catheter presence may also reflect sparse-data effects. Furthermore, because the timing of ICU admission relative to BSI onset was unavailable, the observed association does not establish a temporal or causal relationship between ICU admission and mortality.

The laboratory profile of non-survivors, characterized by higher C-reactive protein and urea together with lower albumin, total protein, platelet count, and several leukocyte subsets, suggests a combined signal of inflammation, catabolic stress, organ dysfunction, and depleted host reserve. In a contemporary haematological malignancy cohort, Wang et al., developed a 30-day mortality prediction model that integrated microbiological and clinical prognostic variables, reinforcing the need to interpret laboratory abnormalities within overall illness severity [31]. Importantly, the lower neutrophil-based ratios among non-survivors should not be misread as protective. In profoundly neutropenic patients, ratios such as the neutrophil-to-lymphocyte ratio may fall because the numerator collapses; their usual interpretation in immunocompetent populations may therefore be reversed or become biologically unstable. Absolute cell counts and clinical context are more informative than isolated ratios in this setting. Furthermore, systemic infections in patients with underlying malignancy frequently trigger severe inflammatory and coagulation cascades. Novel laboratory biomarkers reflecting hypercoagulability and immune-thrombosis, such as the D-Dimer to fibrinogen ratio, have recently been highlighted as useful prognostic markers in cancer-associated sepsis [14]. Although baseline laboratory profiles were recorded in our cohort, serial D-Dimer/fibrinogen ratios were not systematically obtained across all retrospective episodes, representing a valuable target for prospective evaluation [32]. Fungal and polymicrobial episodes accounted for 9.9% and 2.1% of BSIs, respectively. Although these groups were too small for stable adjusted analyses, their presence broadens the empirical and diagnostic challenge, particularly in patients with prolonged neutropenia, extensive prior antibiotic exposure, mucosal injury, or intravascular devices. The clinically relevant implication is not routine indiscriminate broadening of therapy, but repeated risk assessment when fever persists or a patient’s condition deteriorates despite antibacterial treatment. Likewise, the Gram-positive component should remain visible in local protocols: cancer cohorts with vancomycin-resistant enterococcal BSI have demonstrated substantial prior antibiotic exposure and high short-term mortality [33]. Neither annual composite resistance nor annual mortality showed a significant monotonic trend. Stability of the formal trend tests should not be equated with epidemiological constancy. Annual denominators were modest, the composite resistance proportion ranged from 50.0% to 73.3%, and mortality ranged from 9.1% to 37.5%; clinically meaningful changes could therefore remain undetected. The study interval also encompassed the COVID-19 pandemic. Although an exploratory comparison did not identify a statistically significant difference in in-hospital mortality during versus outside the pandemic period, the analysis had limited power and could not account for pandemic-related changes in hospital attendance, admission thresholds, referral patterns, antimicrobial exposure, or case mix. Earlier longitudinal surveillance in haematological malignancy has similarly documented changing pathogen distributions and emerging resistance over time. Continuous, species-level surveillance with standardized resistance definitions is preferable to relying on a single pooled estimate across nine years.

### 4.1. Clinical Implications

These results support a risk-stratified approach to suspected BSI in patients with malignancy. Local empirical regimens should preserve dependable Gram-negative activity and be revised against unit-specific antibiograms, prior colonisation or infection, recent antibiotics, neutropenia depth and duration, and the suspected source. In a large multicentre US cohort, receipt of appropriate initial empirical therapy was associated with lower in-hospital mortality across Gram-negative, Gram-positive, and Candida bloodstream infections [34]. Microbiology workflows should therefore minimise time to identification and susceptibility reporting, with early de-escalation once active, narrower treatment is established. Concurrently, ICU-level instability, severe neutropenia, falling platelet counts, hypoalbuminaemia, and rising inflammatory or renal stress markers should trigger intensified monitoring and early multidisciplinary review. These variables are best used as complementary warning signals rather than as a standalone bedside score, because the present model has not undergone external validation and its apparent discrimination may be optimistic.

### 4.2. Strengths and Limitations

This study has several strengths, including a nine-year observation period, exclusion of likely contaminants, integration of clinical, microbiological, and laboratory variables, restriction of the composite resistance analysis to interpretable bacterial episodes, and an adjusted mortality model. Review of the available records also confirmed that each included episode represented a different patient, supporting the independence assumption used in the analyses. The findings provide institution-specific evidence from a region for which longitudinal cancer-BSI data remain limited. Several limitations require emphasis. First, the retrospective, single-center design limits causal inference and generalizability. Second, the dataset did not record care location at BSI onset or the precise temporal sequence of ICU admission relative to BSI diagnosis; the ICU variable may therefore represent pre-existing critical illness, subsequent sepsis deterioration, or routine postoperative monitoring (as 22.5% of patients had a surgical history). To mitigate this potential mediator and reverse-causality bias, a sensitivity multivariable analysis was performed excluding ICU admission, which confirmed the robust independent prognostic signal of severe neutropenia. Third, constrained by the absolute number of in-hospital deaths (*n* = 39), the multivariable regression model was restricted a priori to four candidate predictors to satisfy the ten-events-per-variable rule and prevent statistical over fitting. Consequently, dynamic downstream laboratory biomarkers of organ dysfunction and inflammation (e.g., CRP, blood urea nitrogen, albumin, and platelet count)-despite showing notable unadjusted differences-were deliberately excluded to avoid severe multicollinearity and over-adjustment bias. The mortality model should therefore be viewed as a parsimonious, exploratory evaluation of baseline clinical features rather than a fully comprehensive predictive scoring framework. Fourth, important treatment-related variables were missing from the dataset. Specifically, information on initial empirical antimicrobial therapy, its appropriateness according to subsequent in vitro susceptibility testing, and the use of routine antibacterial prophylaxis were notably absent. Because inappropriate empirical therapy and delayed active treatment are primary mechanisms through which antimicrobial resistance influences BSI outcomes, the lack of these data represents an important limitation—particularly when interpreting the observed relationships between resistance phenotypes, pathogen distribution, and in-hospital mortality. Fifth, the study period included the COVID-19 pandemic. Although an exploratory comparison found no statistically significant difference in in-hospital mortality during versus outside the pandemic period, modest annual denominators and unmeasured changes in healthcare delivery limit this comparison. Sixth, several multivariable estimates, particularly for ICU and catheter status, were imprecise, and the apparent area under the curve (AUC 0.858) reflects optimistic performance as internal validation (e.g., bootstrapping) or external validation was not performed. The catheter concordance analysis was especially sparse and should be considered strictly descriptive. Seventh, the primary outcome was all-cause in-hospital mortality without a fixed 28-day or 30-day follow-up or formal attribution of infection-specific death. In a cohort where nearly 30% of patients received supportive or palliative care, in-hospital mortality may partly reflect malignant progression or variable length of stay; identified factors thus represent contextual risk markers rather than confirmed BSI-attributable mortality drivers. Eighth, multivariable adjustment for specific malignancy subtypes or chemotherapy protocols was constrained by sample size, leaving underlying disease biology and treatment intensity as potential unmeasured confounders. Ninth, a high proportion of culture-positive episodes ($59.8\%$) were classified as skin contaminants and excluded. Although this stringent exclusion was necessary to maintain high diagnostic specificity and prevent false-positive distortion in risk and mortality models, it reflects the high intensity of blood culture sampling and high prevalence of solitary coagulase-negative staphylococcal isolation in cancer patients. While this filtering may restrict generalizability to true BSI episodes, it ensures that the identified clinical signals apply strictly to genuine bloodstream infections rather than transient line or skin colonizers.

## 5. Conclusions

In conclusion, clinically significant BSI in patients with malignancy at our center was characterized by persistent Gram-negative predominance, a frequent but heterogeneous study-defined resistance composite, and substantial in-hospital mortality. Severe neutropenia and ICU admission were identified as key contextual markers associated with mortality. However, because the primary outcome was all-cause in-hospital mortality in a cohort where nearly 30% of patients received palliative or supportive care, these factors reflect overall host vulnerability and illness severity rather than established causal drivers of infection-attributable death and their magnitudes require cautious interpretation. These findings favor locally adapted empirical therapy combined with rapid microbiological clarification, early recognition of physiological deterioration, and structured source assessment. Prospective multicentre studies incorporating fixed 30-day mortality endpoints, species-level resistance phenotypes, antimicrobial timing and appropriateness, source control, and validated severity scores are needed to determine which modifiable processes can most effectively reduce BSI-related mortality.

## Figures and Tables

**Figure 1:**
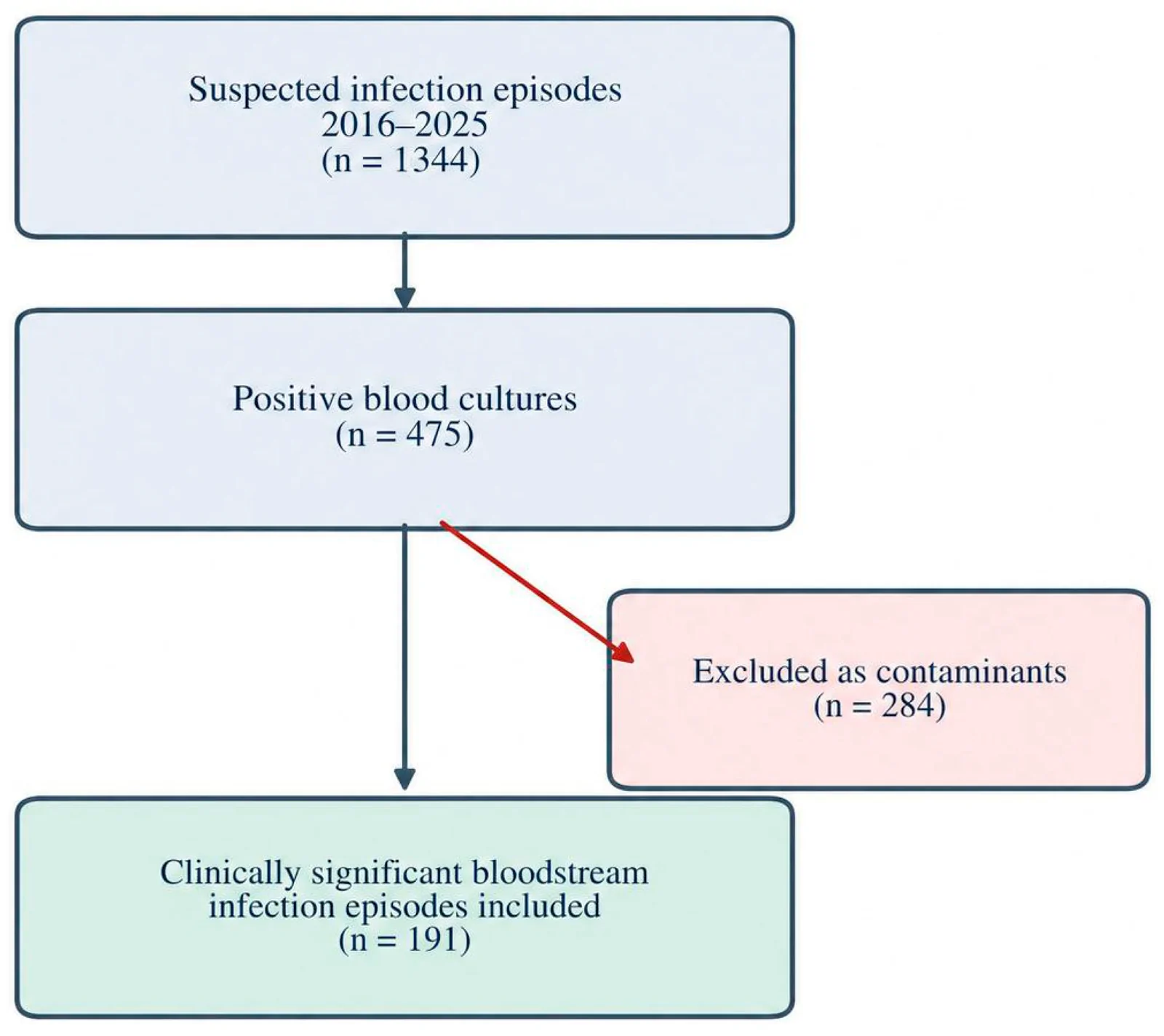
Study flow diagram for the selection of clinically significant bloodstream infection episodes. Of 1344 suspected infection episodes assessed between 2016 and 2025, 475 had positive blood cultures. After 284 episodes classified as contaminants were excluded following clinical and microbiological review, 191 clinically significant bloodstream infection episodes were included in the analytic cohort.

**Figure 2:**
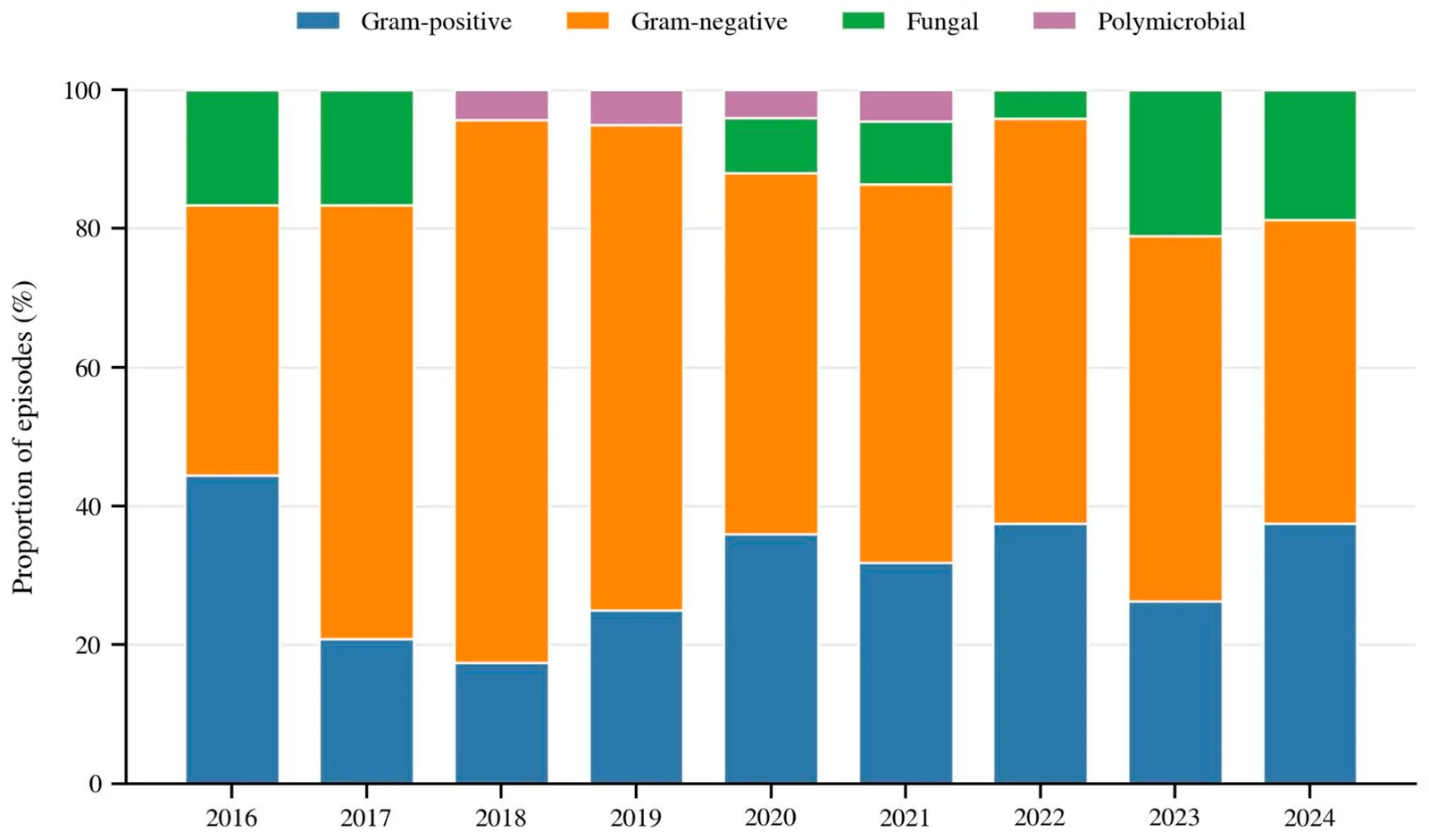
Annual distribution of bloodstream pathogen classes from 2016 to 2024. The 100% stacked bars show the within-year proportions of clinically significant bloodstream infection episodes classified as Gram-positive bacterial, Gram-negative bacterial, fungal, or polymicrobial. Each bar represents all eligible episodes recorded in the corresponding calendar year. (Note: January 2025 [*n* = 2] is excluded from annual trend plotting due to incomplete year coverage but is retained in total cohort analyses.

**Figure 3:**
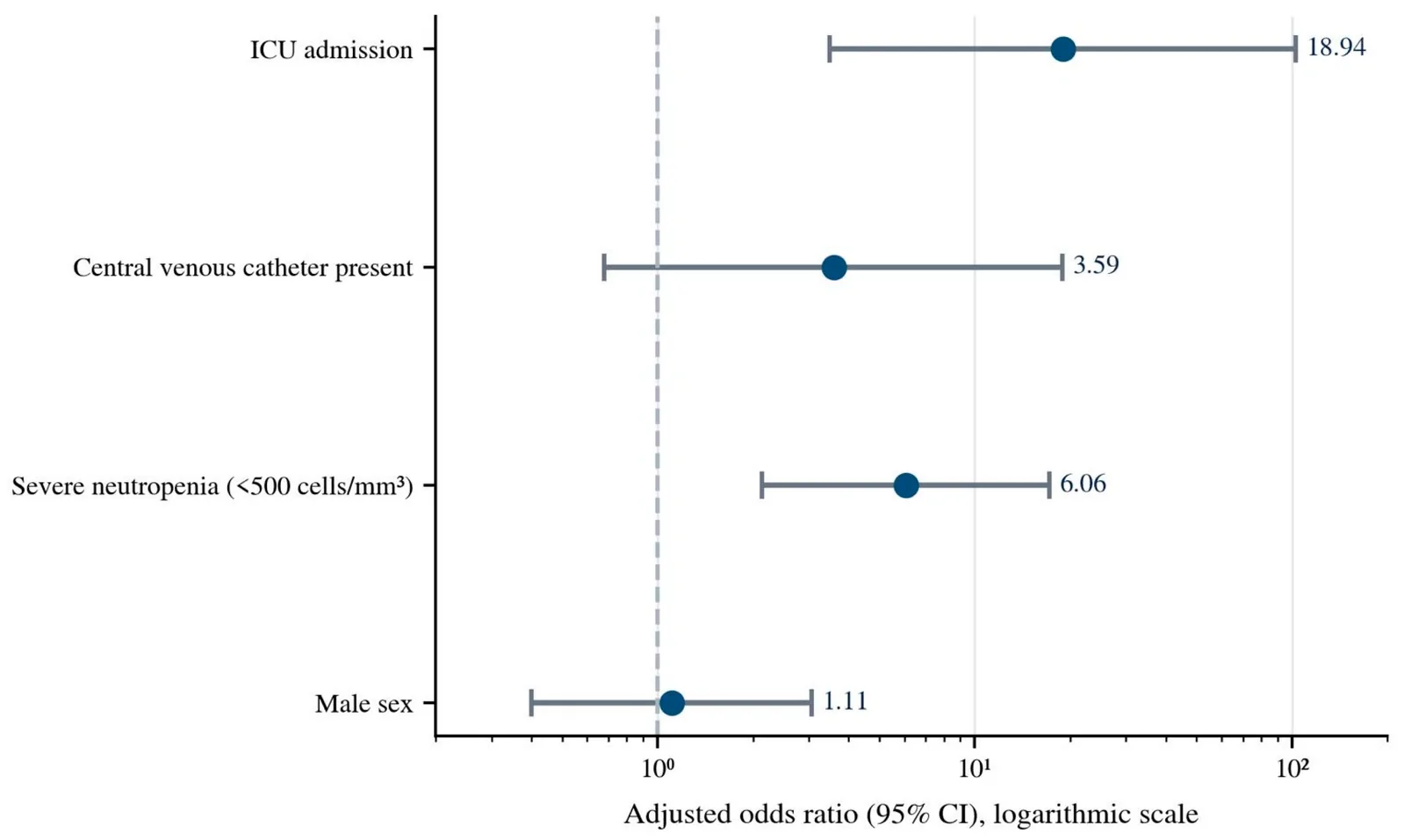
Adjusted associations with all-cause in-hospital mortality. The forest plot presents adjusted odds ratios and 95% confidence intervals from a multivariable logistic regression model that simultaneously included intensive care unit admission, central venous catheter presence, severe neutropenia (<500 cells/mm^3^), and male sex. The reference categories were no intensive care unit admission, no central venous catheter, no severe neutropenia, and female sex, respectively. Points indicate adjusted odds ratios, horizontal bars indicate 95% confidence intervals, and the dashed vertical line denotes the null value of 1.0. CI, confidence interval; ICU, intensive care unit.

**Figure 4:**
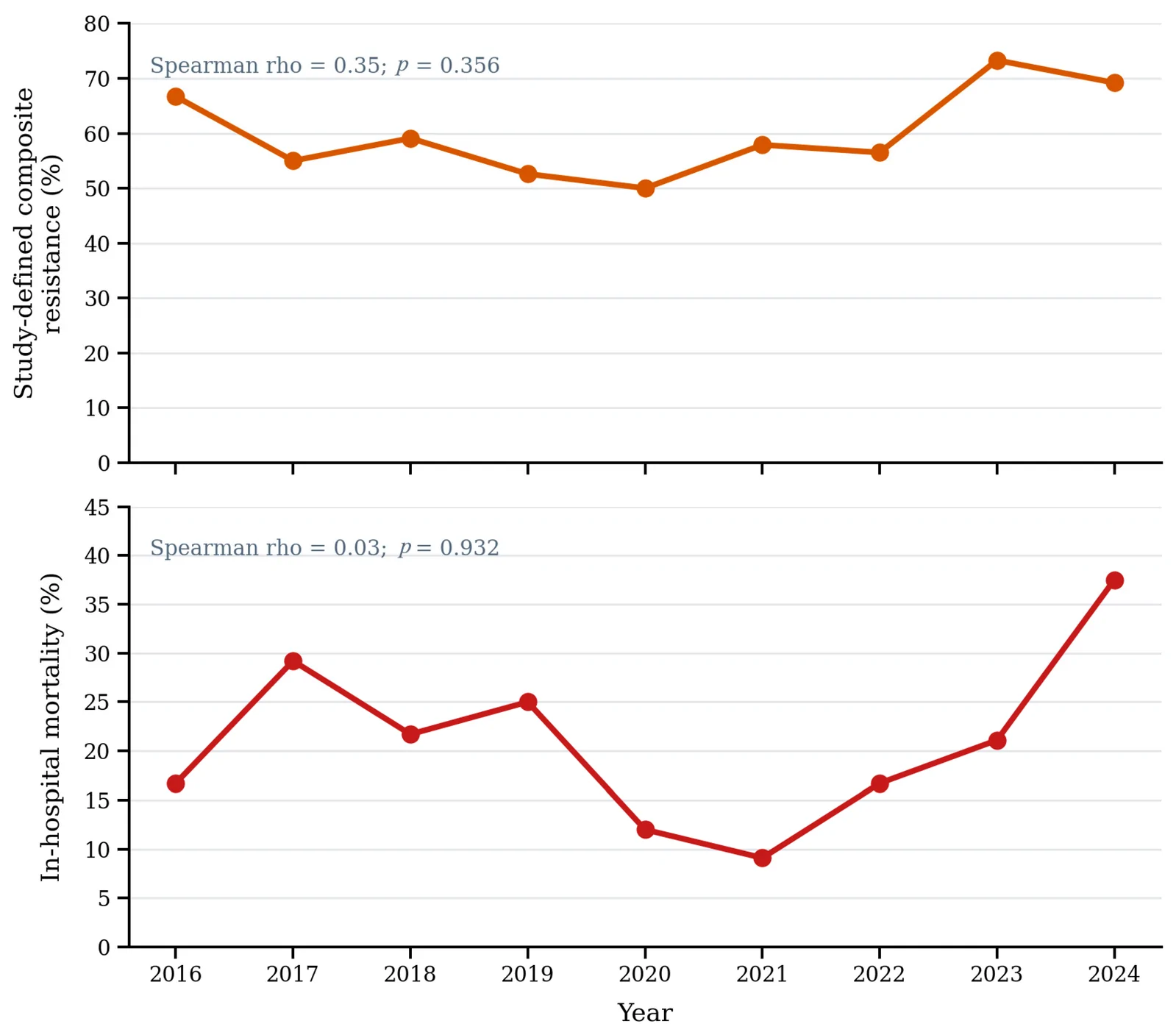
Annual proportions of resistant bacterial isolates and all-cause in-hospital mortality from 2016 to 2024. The upper panel shows the proportion of resistant isolates among episodes with interpretable bacterial antimicrobial susceptibility results. The lower panel shows the proportion of clinically significant bloodstream infection episodes resulting in in-hospital death. Monotonic temporal trends were evaluated using *Spearman* rank correlation. No significant temporal trend was identified for resistance (*Spearman ρ* = 0.35; *p* = 0.356) or in-hospital mortality (*Spearman ρ* = 0.03; *p* = 0.932).

**Table 1:** Baseline demographic and clinical characteristics, overall and according to survival status.

Characteristic	Overall	Survivors	Non-Survivors	*p* Value
Male sex	95 (49.7)	69 (45.4)	26 (66.7)	0.020
Acute leukemia	70 (36.6)	53 (34.9)	17 (43.6)	0.190
Lymphoma	35 (18.3)	25 (16.4)	10 (25.6)	
Plasma-cell disorder	38 (19.9)	34 (22.4)	4 (10.3)	
Chronic leukemia	15 (7.9)	11 (7.2)	4 (10.3)	
Benign hematologic disease/solid tumor	33 (17.3)	29 (19.1)	4 (10.3)	
Diabetes mellitus	28 (14.7)	24 (15.8)	4 (10.3)	0.457
Chronic obstructive pulmonary disease	11 (5.8)	8 (5.3)	3 (7.7)	0.699
Chronic kidney disease	13 (6.8)	10 (6.6)	3 (7.7)	0.731
Hypertension	57 (29.8)	49 (32.2)	8 (20.5)	0.174
Coronary artery disease	20 (10.5)	17 (11.2)	3 (7.7)	0.770
Atrial fibrillation	11 (5.8)	9 (5.9)	2 (5.1)	1.000
Chronic liver disease	1 (0.5)	1 (0.7)	0 (0)	1.000
Concurrent oncologic malignancy	10 (5.2)	7 (4.6)	3 (7.7)	0.429
Neurologic disease	5 (2.6)	5 (3.3)	0 (0)	0.585
History of surgery	43 (22.5)	34 (22.4)	9 (23.1)	1.000
Central venous catheter present	27 (14.1)	7 (4.6)	20 (51.3)	<0.001
ICU admission	25 (13.1)	4 (2.6)	21 (53.8)	<0.001
Severe neutropenia (<500 cells/µL)	72 (37.7)	45 (29.6)	27 (69.2)	<0.001
Supportive/palliative care	57 (29.8)	43 (28.3)	14 (35.9)	0.288
Targeted therapy	4 (2.1)	3 (2)	1 (2.6)	
Multiple-myeloma regimen	40 (20.9)	37 (24.3)	3 (7.7)	
Hypomethylating regimen	36 (18.8)	27 (17.8)	9 (23.1)	
Standard combination chemotherapy	33 (17.3)	27 (17.8)	6 (15.4)	
High-intensity chemotherapy	21 (11.0)	15 (9.9)	6 (15.4)	
Number of chemotherapy courses	2 [0–6]	2 [0.8–6]	2 [0–6]	0.483
Ward length of stay, days	8.0 [5.0–14.0]	8.0 [5.0–13.2]	9.5 [5.0–18.0]	0.295

Data are *n* (%) or median [IQR]. Percentages are column percentages. *p* values were calculated using *Fisher’s* exact test for binary variables, the chi-square test for multi-category variables, and the *Mann–Whitney U* test for continuous variables. ICU, intensive care unit; IQR, interquartile range.

**Table 2:** Infection-related and microbiological characteristics according to survival status.

Characteristic	Overall	Survivors	Non-Survivors	*p* Value
No identified focus	7 (3.7)	4 (2.6)	3 (7.7)	0.038
Primary bloodstream	135 (70.7)	103 (67.8)	32 (82.1)	
Catheter-related	7 (3.7)	6 (3.9)	1 (2.6)	
Respiratory	15 (7.9)	12 (7.9)	3 (7.7)	
Urinary	27 (14.1)	27 (17.8)	0 (0)	
Gram-positive	58 (30.4)	50 (32.9)	8 (20.5)	0.296
Gram-negative	110 (57.6)	84 (55.3)	26 (66.7)	
Fungal	19 (9.9)	14 (9.2)	5 (12.8)	
Polymicrobial	4 (2.1)	4 (2.6)	0 (0)	
Resistant bacterial isolate	99 (58.9)	77 (57.5)	22 (64.7)	0.559
Positive catheter culture ^a^	4 (14.8)	3 (42.9)	1 (5)	0.042
ICU length of stay, days ^b^	3.0 [2.0–7.0]	3.5 [2.0–7.5]	3.0 [2.0–7.0]	0.762

Data are *n* (%) or median [IQR]. The resistant-isolate analysis included 168 episodes with interpretable bacterial susceptibility results (134 survivors and 34 non-survivors). The catheter-culture row included the 27 catheterized episodes (7 survivors and 20 non-survivors). *p* values were calculated using the chi-square test for multi-category variables, *Fisher’s* exact test for binary variables, and the *Mann–Whitney U* test for ICU length of stay. ^b^ ICU length of stay was calculated exclusively among the 25 ICU-admitted episodes (4 survivors and 21 non-survivors). *Sparse*-category comparisons should be interpreted cautiously. ICU, intensive care unit. The resistant-isolate variable was a study-defined heterogeneous composite of organism-specific resistance phenotypes and should not be interpreted as an MDR classification or prevalence estimate. ^a^ Positive catheter culture among catheterized episodes.

**Table 3:** Laboratory findings, overall and according to survival status.

Laboratory Parameter	A (*n*)	Overall	Survivors	Non-Survivors	*p* Value
Neutrophil count, cells/µL	189	1400 [130–4430]	2200 [252–5168]	120 [10–1220]	<0.001
Platelet count, cells/µL	191	38,000 [17,000–108,000]	44,000 [20,750–130,000]	17,000 [12,000–38,500]	<0.001
Lymphocyte count, cells/µL	189	560 [240–1160]	710 [315–1285]	270 [50–655]	0.001
Monocyte count, cells/µL	189	280 [50–780]	350 [70–885]	80 [10–335]	0.004
Eosinophil count, cells/µL	189	10 [0–40]	10 [0–60]	0 [0–0]	<0.001
Hemoglobin, g/dL	191	8.8 [7.8–9.9]	8.8 [7.8–9.9]	8.7 [7.2–9.9]	0.385
Urea, mg/dL	191	45 [29.3–67.2]	39.7 [27.3–64.7]	55.6 [44–77.2]	<0.001
Creatinine, mg/dL	191	0.89 [0.67–1.37]	0.88 [0.66–1.33]	0.95 [0.75–1.83]	0.269
Total protein, g/L	190	55.9 [49.1–63.7]	56.3 [51.1–64.2]	50.1 [47.3–57.3]	<0.001
Albumin, g/L	191	30.4 [27–35]	31.0 [27.5–35.5]	27.6 [24.1–30.8]	<0.001
C-reactive protein, mg/L	188	126.1 [35.3–237.6]	100.2 [30.4–225.8]	193.8 [118.7–313.9]	0.002
Procalcitonin, ng/mL	116	1.64 [0.4–9.34]	1.57 [0.40–8.77]	3.61 [0.43–38.16]	0.174
LDH-to-lymphocyte ratio	179	0.43 [0.2–0.88]	0.38 [0.18–0.73]	0.77 [0.35–3.73]	0.001
Eosinophil-to-lymphocyte ratio	179	0 [0–0.05]	0.02 [0–0.06]	0 [0–0]	<0.001
Neutrophil-to-albumin ratio	191	45.16 [4.09–135.09]	67.01 [7.36–155.41]	3.83 [0.36–47.92]	<0.001
Neutrophil-to-lymphocyte ratio	179	2.44 [0.34–6.36]	2.65 [0.75–7.34]	0.63 [0.04–3.86]	0.001

Values are median [IQR] and were compared with the *Mann–Whitney U* test. A: Available *n* indicates the number of non-missing observations in the full cohort. LDH, lactate dehydrogenase; IQR, interquartile range.

**Table 4:** Multivariable logistic regression model for factors associated with in-hospital mortality.

Associated Variable	Unadjusted OR (95% CI)	*p* Value (Unadjusted)	Adjusted OR (95% CI)	*p* Value (Adjusted)
ICU admission	43.17 (13.32–139.90)	<0.001	18.94 (3.49–102.82)	<0.001
Central venous catheter present	21.80 (8.15–58.37)	<0.001	3.59 (0.68–18.89)	0.132
Severe neutropenia (<500 cells/µL)	5.35 (2.49–11.49)	<0.001	6.06 (2.13–17.23)	<0.001
Male sex	2.41 (1.15–5.03)	0.020	1.11 (0.40–3.06)	0.840

The multivariable model included all four predictors shown (*n* = 191; 39 deaths). Model likelihood-ratio chi-square = 74.32 (4 df), *p* < 0.001; Akaike information criterion = 129.03; McFadden pseudo-R2 = 0.384; apparent area under the receiver operating characteristic curve = 0.858. CI, confidence interval; ICU, intensive care unit; OR, odds ratio.

## Data Availability

The data that support the findings of this study are available from the corresponding author upon reasonable request.

## Supplementary Materials

The following supporting information can be downloaded at: https://www.mdpi.com/article/10.3390/diagnostics16203314/s1, Table S1: Concordance between paired blood and catheter culture findings. Only catheterized episodes with a positive catheter culture are shown. CoNS, coagulase-negative *staphylococci*. Table S2: Annual distribution of pathogen classes, bacterial resistance, and in hospital mortality.
