# Supplementary material for: Bloodstream Infections in Patients with Malignancy: Microbiological Epidemiology, Antimicrobial Resistance, and Factors Associated with Mortality in a Nine-Year Single-Center Retrospective Cohort
Source: Diagnostics (Basel). 2026 Oct 12;16(20):3314. doi: 10.3390/diagnostics16203314 (PMC13703511; doi:10.3390/diagnostics16203314)
Supplement: Supplementary file 1 [file diagnostics-16-03314-s001.zip › diagnostics-4567107-supplementary.pdf]

Supplementary Table S1. Concordance between paired blood and catheter culture findings. Only catheterized episodes with a positive catheter culture are shown. CoNS, coagulase-negative *staphylococci*.

Study record	*Blood pathogen class*	*Catheter pathogen class*	*Class concordance*	*Blood susceptibility category*	*Catheter susceptibility category*	*Susceptibility concordance*
10	*Gram-negative*	*Gram-negative*	*Yes*	*Susceptible Gram-negative isolate*	*Susceptible Gram-negative isolate*	*Yes*
12	*Gram-negative*	*Gram-negative*	*Yes*	*Resistant Gram-negative isolate*	*Resistant Gram-negative isolate*	*Yes*
78	*Gram-positive*	*Gram-positive*	*Yes*	*Methicillin-resistant CoNS*	*Methicillin-resistant CoNS*	*Yes*
178	*Gram-negative*	*Gram-negative*	*Yes*	*Resistant Gram-negative isolate*	*Resistant Gram-negative isolate*	*Yes*

Supplementary Table S2. Annual distribution of pathogen classes, bacterial resistance, and in hospital mortality.

Year	*Episodes n*	*Gram-positive n (%)*	*Gram-negative n (%)*	*Fungal n (%)*	*Poly-microbial n (%)*	*Resistant bacterial isolates n/N (%)*	*Deaths n (%)*
2016	*18*	*8 (44.4)*	*7 (38.9)*	*3 (16.7)*	*0 (0)*	*10/15 (66.7)*	*3 (16.7)*
2017	*24*	*5 (20.8)*	*15 (62.5)*	*4 (16.7)*	*0 (0)*	*11/20 (55)*	*7 (29.2)*
2018	*23*	*4 (17.4)*	*18 (78.3)*	*0 (0)*	*1 (4.3)*	*13/22 (59.1)*	*5 (21.7)*
2019	*20*	*5 (25)*	*14 (70)*	*0 (0)*	*1 (5)*	*10/19 (52.6)*	*5 (25)*
2020	*25*	*9 (36)*	*13 (52)*	*2 (8)*	*1 (4)*	*11/22 (50)*	*3 (12)*
2021	*22*	*7 (31.8)*	*12 (54.5)*	*2 (9.1)*	*1 (4.5)*	*11/19 (57.9)*	*2 (9.1)*
2022	*24*	*9 (37.5)*	*14 (58.3)*	*1 (4.2)*	*0 (0)*	*13/23 (56.5)*	*4 (16.7)*
2023	*19*	*5 (26.3)*	*10 (52.6)*	*4 (21.1)*	*0 (0)*	*11/15 (73.3)*	*4 (21.1)*
2024	*16*	*6 (37.5)*	*7 (43.8)*	*3 (18.8)*	*0 (0)*	*9/13 (69.2)*	*6 (37.5)*

Percentages for pathogen classes and deaths use all episodes in the corresponding year as the denominator. Resistance percentages use only episodes with an interpretable bacterial susceptibility category; denominators are shown as n/N. The resistance variable is a study-defined heterogeneous composite of organism-specific phenotypes and should not be interpreted as an MDR classification or prevalence estimate.
